# Dr. Henry W. Caldwell

**Published:** 1915-06

**Authors:** 


**Dr. Henry W. Caldwell,** Buffalo 1866, died at his home in Pulaski, Feb. 24, aged 74.

